# Viral- and fungal-mediated behavioral manipulation of hosts: summit disease

**DOI:** 10.1007/s00253-024-13332-x

**Published:** 2024-10-23

**Authors:** Abolfazl Masoudi, Ross A. Joseph, Nemat O. Keyhani

**Affiliations:** Department of Biological Sciences, University of Illinois, Chicago, IL USA

**Keywords:** Summit disease, Behavioral manipulation, Entomopathogenic fungi, Baculovirus infection, Host–pathogen interaction, Neurobiological mechanisms

## Abstract

**Abstract:**

Summit disease, in which infected hosts seek heights (gravitropism), first noted in modern times by nineteenth-century naturalists, has been shown to be induced by disparate pathogens ranging from viruses to fungi. Infection results in dramatic changes in normal activity patterns, and such parasite manipulation of host behaviors suggests a strong selection for convergent outcomes albeit evolved via widely divergent mechanisms. The two best-studied examples involve a subset of viral and fungal pathogens of insects that induce “summiting” in infected hosts. Summiting presumably functions as a means for increasing the dispersal of the pathogen, thus significantly increasing fitness. Here, we review current advances in our understanding of viral- and fungal-induced summit disease and the host behavioral manipulation involved. Viral genes implicated in this process include a host hormone-targeting ecdysteroid UDP-glucosyltransferase (apparently essential for mediating summit disease induced by some viruses but not all) and a protein tyrosine phosphatase, with light dependance implicated. For summit disease-causing fungi, though much remains obscure, targeting of molting, circadian rhythms, sleep, and responses to light patterns appear involved. Targeting of host neuronal pathways by summit-inducing fungi also appears to involve the production of effector molecules and secondary metabolites that affect host muscular, immune, and/or neurological processes. It is hypothesized that host brain structures, particularly Mushroom Bodies (no relation to the fungus itself), important for olfactory association learning and control of locomotor activity, are critical targets for mediating summiting during infection. This phenomenon expands the diversity of microbial pathogen-interactions and host dynamics.

**Key points:**

• *Summit disease or height seeking (gravitropism) results from viral and fungal pathogens manipulating insect host behaviors presumably to increase pathogen dispersal.*

• *Insect baculoviruses and select fungal pathogens exhibit convergent evolution in host behavioral manipulation but use disparate molecular mechanisms.*

• *Targets for affecting host behavior include manipulation of host hormones, feeding, locomotion, and immune, circadian, and neurological pathways.*

## Introduction

Insects (*Arthropoda*) are the most populous category of land-dwelling creatures, reigning supreme in sheer numbers and diversity, boasting an estimated 5.5 million distinct species (Perveen et al. 2023). This remarkable order contributes significantly to the natural world by serving, amongst many other roles, as crucial plant pollinators and biological guardians. Insects play vital roles in maintaining the health and balance of almost all terrestrial and aquatic ecosystems, aiding in the recycling of nutrients, and contributing to the food chain and natural resources (Brown and Paxton 2009). Numerous insect species are also considered serious (animal/human/agricultural) pests, acting as vectors of plant and animal disease-causing agents and threatening almost all agricultural crops and staple food resources (Eggleton 2020). Insects such as mosquitoes, lice, fleas, and bed bugs, along with ticks (Acari), vector and transmit a wide array of pathogens, including bacteria, viruses, nematodes, and protozoa, posing substantial risks (and economic costs) to plant and animal health (Wang et al. 2023). Several groups of organisms can infect and kill insects, including viruses, bacteria, protists, nematodes, and fungi (including microsporidia) (Glare et al. 2012; Hajek and Shapiro-Ilan 2017). Such pathogens can invade and reproduce in insect hosts, often spreading the infection to other insects, including as part of enzootic or epizootic infection cycles that can significantly affect (reduce) insect populations (Jaronski 2010; Joseph et al. 2024a). Microbial entomopathogens display varying degrees of specificity; some are highly specific, infecting one or several insect species, while others have broader host ranges, potentially affecting hundreds of species across different orders and even phyla (Elya and De Fine Licht 2021; Wang et al. 2024). These infectious microorganisms fall into four main categories: (i) opportunistic, (ii) potential, (iii) facultative, and (iv) obligate pathogens. Opportunistic pathogens are typically “harmless” microorganisms that can cause disease under specific conditions, such as when the host is sick, or its immunity is compromised, e.g., the fungus, *Aspergillus flavus* (*Eurotiales*, *Aspergillaceae*) infection of larval and adult bees (*Hymenoptera*) (Onstad et al. 2006). Potential pathogens lack a direct method of infecting hosts but can multiply and induce disease if they enter through, for instance, a wound; they often thrive in culture and do not target specific hosts, e.g., the bacterium *Serratia marcescens* (*Enterobacterales*, *Yersiniaceae*). Facultative pathogens can infect and multiply within host animals while also thriving in the environment; they are easily cultured in vitro, e.g., the bacterium *Bacillus thuringiensis* (*Bacillales*, *Bacillaceae*) and Hypocrealean fungi such as *Beauveria* (*Cordycipitaceae*) and *Metarhizium* sp. (*Clavicipitaceae*). Obligate pathogens multiply solely on/within specific hosts, leading to specific disease phenotypes; they typically have a limited host range and are challenging to culture in vitro in the absence of their hosts, necessitating mechanisms for transmission between host generations, e.g., baculoviruses, *Paenibacillus popilliae* (*Bacillales*, *Paenibacillaceae*), microsporidian fungi, and fungi from the *Entomophthorales*. One central characteristic that distinguishes these groups is their point of entry, i.e., where and how the entomopathogen infiltrates or gains access to an insect host (Vega and Kaya 2012). The primary routes through which pathogens enter an insect host usually involve either the mouth (*per os*) and/or the protective outer layer of the body, known as the integument (*per cutaneous*), although infection via the anus and/or spiracles can also occur (Castagnola et al. 2016). After the microbe multiplies within an infected insect host, infective propagules (e.g., viral particles, spores, conidia) are discharged from the still-living or deceased host into the environment to initiate new infections in other vulnerable hosts (Lovett et al. 2020a). Non-obligate pathogens can often grow saprophytically, some form alternate associations with other organisms, e.g., *Beauveria* and *Metarhizium* sp., that can act as epi-/endophytes and/or persist in the rhizosphere, and can have consequences on host immunity and behavior, although not necessarily involving manipulation per se, i.e., impacting pathogen recognition, avoidance, and host sanitation behaviors (Zhang et al. 2024, 2023; Zheng et al. 2023). Many obligate pathogens produce “resting” spores that can persist in the environment (soil) until contact/attachment with a suitable host, which initiates growth and infection, e.g., *Massospora* sp. that are fungal pathogens of cicadas (Macias et al. 2020).

Within this general context, a particular subset of insect microbial pathogens have evolved the ability to influence the behavior (nervous system) of their hosts, altering normal behavior to maximize the fitness of the pathogen. One example involves manipulating the host such that mortality/morbidity occurs in locations, geometries, and/or specific times (of the day) that maximize the spread of the pathogen, e.g., daytime or night, at an elevated height, and with the dead or dying host facing downwards so that the infectious particles can “rain” down on unsuspecting hosts during periods in which healthy targets are most active—summit disease (Elya et al. 2023; Malagocka et al. 2015). Such phenomena are sometimes referred to as pathogen-extended phenotypes, i.e., phenotypes in which gene(s) may affect more than the organism within which it is encoded, but can “extend” to those it associates with (Shang et al. 2015). Here, we review one such behavioral manipulation that has occurred convergently by disparate pathogen groups. Knowledge gained from examining host-modulating diseases such as summit disease will impact our understanding of the range of neurological, circadian, immunological, and even musculatory manipulation that microbial pathogens can exert on hosts.

## Summit disease

**Fig. 1:**
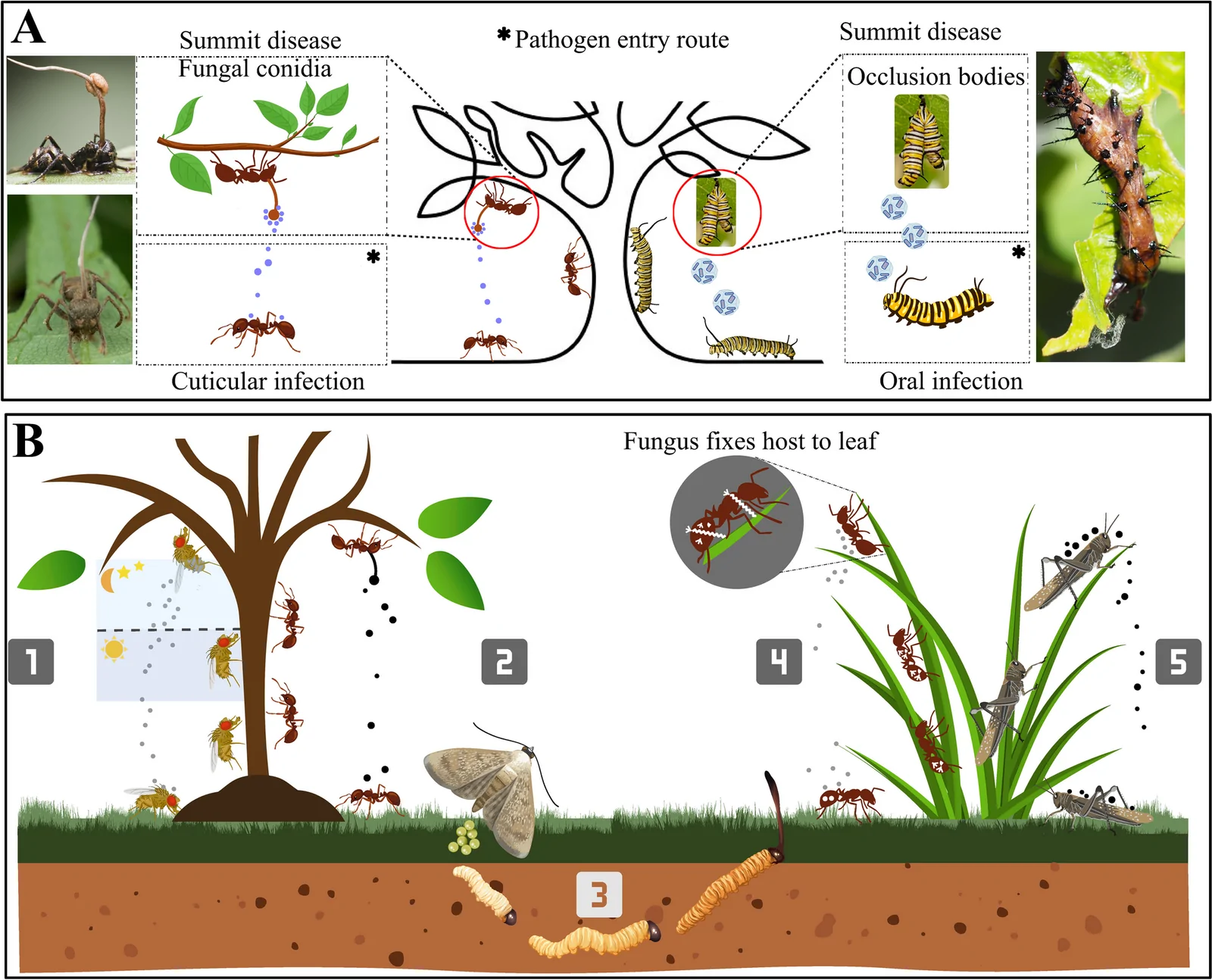
Summit disease benefits pathogen transmission. **A** Baculoviruses primarily infect insects through ingestion (*per os*), whereas entomopathogenic fungi can infect insects by direct penetration of the cuticle (i.e., topical infection, *per cutaneous*) and via ingestion. **B** Examples of fungal infection in different hosts: (1) *Entomophthora muscae* (*Entomophthorales*, *Entomophthoraceae*) infects dipteran insects. Infected flies exhibit a behavior known as summiting (height seeking, gravitropism), ascending to a higher point as the infection progresses. Once the elevated point is reached, the dying fly extends its proboscis, becoming affixed to the substrate via sticky secretions, and then raises its wings. This specific posture facilitates the transmission of the fungus to other potential hosts. Death of the host is timed, with *E. muscae* typically causing host death at sunset. (2) *Ophiocordyceps* (*Hypocreales*, *Ophiocordycipitaceae*)-ant infection system. After fungal spores infect the ant, *Ophiocordyceps* hijacks the central nervous system of the ant, causing it to leave its colony, climb vegetation, and attach itself to a leaf or twig in a characteristic “death grip.” The fungus then consumes the ant’s body, using it as a nutrient source. It eventually produces a spore-producing stalk that emerges from the ant’s body, allowing the fungus to release spores into the environment to infect new hosts. (3) Lepidopteran hosts infected with *O. sinensis*. Insect host nymphs typically burrow and feed underground. Infection results in their displacement upwards to a position a few centimeters below the soil surface. At this point, fungal infection results in death before the host can pupate. The position of the dead cadaver is optimal for the fungal synnema to grow upwards, breaching the soil-air interface to sporulate and complete the fungal life cycle. (4) The fungus *Pandora formicae* (*Entomophthorales*, *Entomophthoraceae*) infects red wood ants (*Formica rufa*, Linnaeus). Infected ants are often found attached to blades of grass, with a noticeable bundle of fungal rhizoids emerging from the ventral side of their thorax. (5) Grasshoppers (*Orthoptera*, Latreille) infected with *Entomophaga grylli* (*Entomophthorales*, *Entomophthoraceae*). Infected hosts typically climb to elevated positions, where the fungus grows from the dead cadaver, producing sporulation structures. Summiting aids in the dispersal of fungal spores. Images sourced from Shutterstock and BioRender

Numerous organisms infect animals and induce them to exhibit specific, unusual behaviors that enhance parasite survival and reproductive success, often at the detriment of the host. For instance, “zombie ant” fungi from the genus *Ophiocordyceps* (*Hypocreales*, *Ophiocordycipitaceae*) manipulate their carpenter ant hosts to leave their nests and deviate from their usual foraging on the surface or surrounding soil to climb nearby plants or twigs, and, in their final moments, latch onto vegetation where they eventually die at elevated locations (Fig. 1) (Hughes et al. 2011). After a few days, a fungal stalk protrudes from the pronotum of the deceased ant, which is apparently ideally positioned to distribute spores onto unsuspecting ants below (Bekker et al. 2021). More broadly, this fits into other examples of behavioral manipulation, including the jewel wasp (*Ampulex compressa*, Fabricius) that incapacitates the American cockroach (*Periplaneta americana*, Linnaeus) (Rana et al. 2023); protozoans, particularly *Toxoplasma gondii* (*Eucoccidiorida*, *Sarcocystidae*), that dull rodent instinctive fear of cats (Boillat et al. 2020); and hairworms (phylum *Nematomorpha*) that drive crickets (order *Orthoptera*) to jump into water, ultimately leading to their demise, that demonstrate how parasites can manipulate host behavior to increase their survival, propagation, and/or transmission (Cunha et al. 2023).

Summit disease, also known as tree-top disease or Wipfelkrankheit, was first reported in modern times in Germany in the late nineteenth century as a behavioral manipulation seen in insects parasitized by specific pathogens or parasites (Hofmann 1891). This phenomenon causes infected insects to climb to higher elevations, such as the tops of plants or trees, where they eventually die, and, as mentioned, with the end result of increasing the dispersal of spores when the host dies (Fig. 1) (Bhattarai et al. 2018b). Summit disease can be triggered by a variety of parasites, including viruses, fungi, and even trematodes (Rand et al. 2023) and certain types of parasitic wasps, impacting a broad spectrum of insects, with particular targets well characterized within ants, beetles, crickets, caterpillars, and flies, but even potentially extending arachnids such as spiders (Elya et al. 2023; Joseph et al. 2024b; Malagocka et al. 2015). Summit disease represents an example of how parasites can exert profound control over their hosts, often altering natural behaviors to enhance their own survival and reproductive success. The most common symptom of summit disease is positioning of the host at a higher elevation just before death (Lovett et al. 2020a; Will et al. 2023). This behavior is considered to provide several benefits to the parasite: first, it can increase the visibility of the dying host, making it more likely to be consumed by a predator, which then acts as the next host and/or carrier/dispersal agent for the pathogen. One example is the infection of host ants by the trematode, *Dicrocoelium dendriticum*, where infected ants exhibit altered diurnal activities, including nocturnal climbing behavior such as ascending vegetation during the nighttime (Gasque and Fredensborg 2023). This climbing behavior increases the likelihood of predation by grazing mammals, which in turn act as the next host for the parasite, allowing for the completion of the parasite’s life cycle. As mentioned previously, the second potential broad benefit to the parasite of summit behavior is that it positions the dying host for optimal dispersal of the next generation of parasite infectious propagules, e.g., spores, occlusion bodies. Examples include baculovirus infection of lepidopteran and other insect larvae (Bhattarai et al. 2018b; Llopis-Giménez et al. 2022) and fungal infection of a range of insects (Will et al. 2023). Microorganisms, including bacteria, fungi, protists, viruses, baculoviruses, and a subset of entomopathogenic fungi, have been characterized as the main agents causing summit disease. Here, we seek to summarize mechanisms of summit disease caused by fungi and viruses, identifying shared traits and unique characteristics to help develop models for understanding why a specific group of microbial pathogens is linked to summit disease. Correlations may be attributed to the production of microbial toxins, which can encompass various classes, including pore-forming toxins, insect ion channel modulators, and psychoactive compounds amongst others (Chalivendra 2021). It is likely that combinations of neuroactive, immunomodulatory, and metabolic compounds function to facilitate the manipulation of insect behavior during summit disease. If true, microbial insect pathogens capable of generating psychoactive and other compounds could be designated as parasitic modulators, offering a conceptual framework to comprehend the mechanisms underlying summit disease. Parasites adapted to control behavior serve as distinct evolutionary experiments, wherein genes from both the host and parasite govern and affect neural processes within the brain (Hughes and Libersat 2019).

## Baculoviruses: influencing host physiology and behavior

**Fig. 2:**
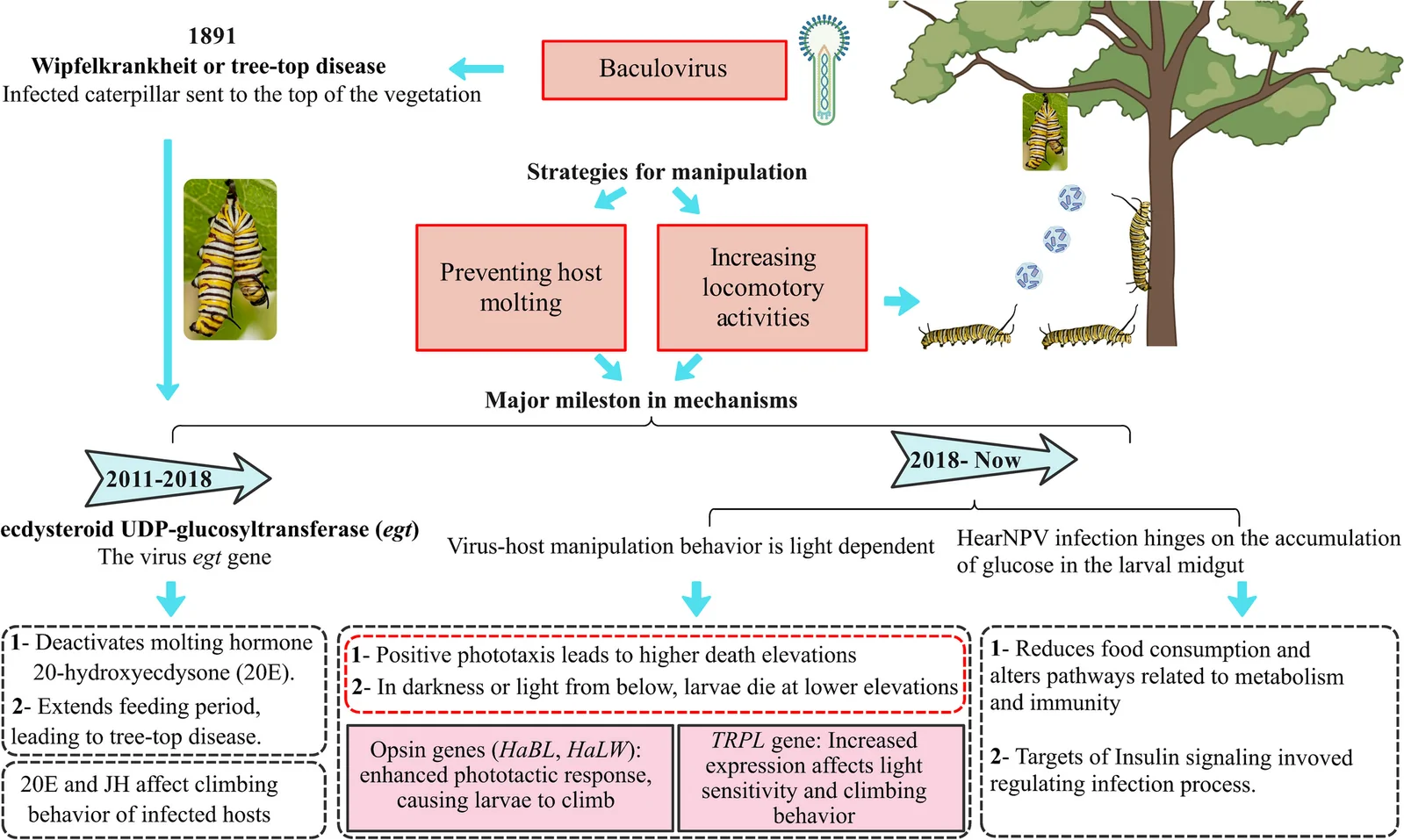
Basic milestones in understanding baculovirus manipulation of insect hosts

The *Baculoviridae* family encompasses a broad range of double-stranded DNA viruses, e.g., nucleopolyhedroviruses (NPVs) and granuloviruses, which target insects and are noted for being able to infect over 800 insect species (Liu et al. 2022). Baculoviridae are separated into four genera: *Alphabaculovirus* and *Betabaculovirus*, predominantly found targeting *Lepidoptera*; *Gammabaculovirus*, primarily infecting *Hymenoptera*; and *Deltabaculovirus*, commonly associated with *Diptera* (Nagamine 2022). Baculoviruses infect insects *per os*, i.e., by being ingested in the form of occlusion bodies, which dissolve in the insect midgut, releasing occlusion-derived virions (ODVs). These ODVs then infect midgut epithelial cells. The virus replicates and spreads to other tissues, often using viral fibroblast growth factors to degrade barriers, facilitating systemic infection (Gelaye and Negash 2023). Baculoviruses employ several strategies to manipulate the physiology and behavior of their host insects, thereby optimizing virus replication and spread (Fig. 2). These strategies include preventing host molting and increasing locomotory activities (Liu et al. 2022). By halting the molting process, the virus extends the feeding period of the host, enhancing viral proliferation. Additionally, these viruses induce: (i) increased activity and movement of the host and (ii) in infected hosts seeking heights (gravitropism), i.e., climbing upwards on various vegetation (“summiting”), where they eventually die, the latter behavior presumably facilitating the dissemination of the virus (Hofmann 1891; Wang and Hu 2019). Summiting benefits the virus by increasing viral dispersal to lower vegetation and also potentially by increasing visibility/predation by birds, which may help in the further/wider spread of viral occlusion bodies. Baculoviruses were the initial parasites recognized to demonstrate “genes for the extended phenotype,” as described by Dawkins (1982), i.e., genes that exhibit phenotypic effects in host organisms, and their infection of caterpillars has been recognized as an ideal model for exploring how parasites trigger behavioral changes in their hosts (Liu et al. 2022; Ros et al. 2015). Early studies examining European gypsy moth (*Lymantria dispar*, Linnaeus) larvae infected with nucleopolyhedrovirus, or LdMNPV, revealed distinctive behaviors just before death (D'Amico and Elkinton 1995); whereas healthy *L. dispar* larvae take refuge in bark crevices or move down to the soil during daylight hours to avoid predation by birds, venturing onto leaves to feed at night, baculovirus-infected larvae climb to the apex of host trees, where they die and liquefy, dispersing millions of infectious virus particles. Such “tree top disease” in *L. dispar* is triggered by the activation of the baculovirus gene encoding for ecdysteroid uridine 5′-diphosphate (UDP)–glucosyltransferase (*egt*) ((Hoover et al. 2011). The *egt* gene product deactivates the insect developmental (molting) hormone, 20-hydroxyecdysone (20E), by adding a carbohydrate moiety from a nucleotide donor to the hydroxyl group on 20E (glucosylation), resulting in the production of 20E-glucoside (O'Reilly et al. 1992). 20E has a broad range of functions, including affecting immunity, and under normal conditions, it can cross the blood–brain barrier, reaching the central nervous system (CNS), where it impacts the production of other developmental hormones, the regulation of nocturnal circadian rhythm, and likely other neurological processes, ultimately acting not only to induce molting and pupation but to do so in locations/conditions that optimize the survival of the organism (Adamo 2019). It is important to note that pupation is not directly caused by 20E alone but occurs when a decrease in the levels of juvenile hormone allows 20E to trigger the metamorphic process (Gelman et al. 2007; Truman 2019). Thus, by reducing (active) 20E levels, baculoviruses reduce/inhibit these normal host behaviors to increase their own fitness. Comparable impacts of a virally encoded *egt* on mediating tree-top/summit disease were also noted in larvae of the beet armyworm (*Spodoptera exigua*, Hübner) infected with *S. exigua* nucleopolyhedrovirus (SeMNPV) (Han et al. 2015a), where infection prolongs the host life span and extends its feeding period, keeping the host in a continuously active feeding state. Genomic analyses have shown that the *egt* gene is present in nearly all lepidopteran baculoviruses except one specific clade of granuloviruses (Ahn et al. 2012), which may have been acquired via horizontal gene transfer from their insect hosts (Wang and Hu 2019). However, during infection of the cabbage looper (*Trichoplusia ni*, Hübner) and *S. exigua* by the *Autographa californica* multiple nucleopolyhedrovirus (AcMNPV), the *egt* gene is not required for summiting, suggesting either a lack of a conserved role for the *egt* gene in inducing summit disease and/or the requirement of other factors (Ros et al. 2015). Climbing behavior in these larvae seems to be linked to molting rather than other biological processes. RNA interference (RNAi) knockdown of viral *egt* expression has been shown to reduce virus-induced summit disease in cotton bollworm (*Helicoverpa armigera*, Hübner) larvae infected with HearNPV (Zhang et al. 2018). In this system, not only are 20E levels decreased but juvenile hormone (JH) titers have been shown to increase. Although the exact mechanism by which JH titers are modulated in response to viral infection remains unclear, it is known that periodic releases of ecdysone stimulate molting, and that JH is released at every molt. JH levels typically decrease as larvae develop, and when JH levels decrease below a certain threshold, pupation occurs, with additional epi-endocrinological events likely occurring (De Loof et al. 2013). High levels of JH, however, can maintain larval characteristics by preventing the appearance of adult traits. These findings suggest that *egt*-induced summit disease may be an indirect consequence of 20E influencing molting processes. Intriguingly, the noctuid-specific fungal insect pathogen, *Metarhizium* (formerly *Nomuraea*) *rileyi*, also targets host 20E levels; however, the fungal enzyme is an ecdysteroid 22-oxidase which inactivates the hormone-preventing molting and dampening immune activation, but infection does not seem to result in summit disease (Zhu et al. 2021).

Although viral *egt* appears critical in some cases, this does not appear to be universal, and other mechanisms/genes may be involved in mediating summit disease in different insect viruses. Such findings suggest that manipulation of host behavior by baculoviruses might have evolved repeatedly and/or functions as an adaptation that exploits the host’s natural responses to hormonal signals (Hoover 2019). Another baculovirus gene, protein tyrosine phosphatase (*ptp*), which acts as a virion structural protein, appears to also facilitate infection of host neuronal tissues, subsequently enhancing locomotory activity, inducing climbing behavior in infected caterpillars (Gasque et al. 2024). Likely a similar (to *egt*) example of acquisition from an ancestral host via horizontal gene transfer, *ptp* may substitute and/or function in concert with *egt* in promoting the increased movement and climbing behavior observed in baculovirus-infected silk moths (*Bombyx mori*, Linnaeus) (Katsuma et al. 2012). However, the presence of *egt* and *ptp* varies across different baculovirus species, with some having both, one, or neither of these genes (Han et al. 2015a; O’Reilly 1997). These findings indicate that additional as yet unknown mechanisms exist in some viruses, that ultimately result in the same extended phenotype as those that use an apparent *egt*/*ptp* mediated mechanism. Daylight can play a critical role in the manifestation of baculovirus-induced summit disease (Bhattarai et al. 2018a; Gasque et al. 2019; Van Houte et al. 2015). Third instar larvae of *S. exigua* infected with SeMNPV, *H. armigera* with HearNPV, and *L. dispar* with LdMNPV demonstrate an attraction to light (positive phototaxis), likely acting to promote summiting/elevation seeking due to illumination from above. This is supported by experiments where infected larvae died at lower elevations when placed in total darkness or when illuminated from below, whereas uninfected larvae exhibited no light preference, showing similar movement patterns in both light and dark environments. Interestingly, not all virus-host interactions result in light-dependent behavior; AcMNPV-infected *T. ni* larvae have been reported to die at elevated positions even in constant darkness (Han et al. 2015b), suggesting a behavior known as negative geotaxis may be involved in this particular pairing, again indicating that different baculoviruses may employ distinct strategies to manipulate host behavior. HearNPV has been shown to manipulate the visual perception of their caterpillar hosts, i.e., *H. armigera*, resulting in increased phototaxis, which induces climbing to elevated positions (Liu et al. 2022). This climbing behavior predominantly occurs during daylight and can be manipulated by the distance between the infected insect and the light source. Infection results in the increased expression of the host opsin (light receptor) genes, *HaBL* and *HaLW*, whose protein products respond to blue light and long-wave light, respectively, as well as the transient receptor potential-like channel (*TRPL*) gene, proving a mechanism for the light-induced summiting behavior. HearNPV infection has also been shown to result in host metabolic reprogramming via the insulin signaling pathway (Tian et al. 2023). Infected larvae treated with insulin resulted in reduced viral replication and climbing behavior. Conversely, suppression of the insulin receptor gene resulted in increased climbing behavior. In addition, inhibition of glycolysis using dichloroacetate (DCA) enhanced viral replication but reduced climbing behavior, whereas inhibition of the tricarboxylic acid (TCA) cycle did not significantly affect climbing behavior. Furthermore, dietary restriction led to decreased viral replication and reduced climbing behavior, while glucose supplementation had the opposite effect, promoting both climbing behavior and viral replication. These findings provide mechanistic links between metabolic processes and the increased locomotion and subsequent climbing behavior seen during these infections.

Neuropeptides, small protein-like molecules that function as neurohormones, neurotransmitters, and neuromodulators, are essential components of the central nervous system (CNS), regulating insect physiology and behavior (Zup et al. 2022). It is not too surprising that some of these molecules are targeted by baculoviruses as part of their host manipulation strategy. SeMNPV infection of *S. exigua* larvae has been shown to decrease expression of the neuropeptide, proctolin, involved in modulating muscle contractions and motor functions, which are crucial for the larvael movement and other physiological processes (Llopis-Giménez et al. 2022). In *S. exigua* larvae, AcMNPV particles are detectable in the CNS as early as 3 days post-infection (Gasque et al. 2024). In this instance, the viral *ptp* gene implicated in mediating summit disease does not appear to be required for CNS infection. Instead, the virus enters the CNS via the trachea, spreading from the caudal to the frontal regions, progressing from the outer CNS cell layers towards the inner cell layers, independent of *ptp* involvement (Gasque et al. 2024). A summary of the major viral insect pathogens resulting in summit disease is included in Table 1.

**Table 1:** Key aspects of viral and fungal pathogens leading to summit disease

Pathogen	Host	Note on infection: gene(s)/effector(s) implicated	Proposed host target hormone/pathway	Reference(s)
Virus				
*Lymantria dispar* Nucleopolyhedrovirus (LdMNPV)	European gypsy moth (*Lymantria dispar*, Linnaeus)	The *egt* gene (Ecdysteroid uridine 5′-diphosphate (UDP)–glucosyltransferase)	Alters molting hormone (20-hydroxyecdysone) affects circadian rhythm, induces climbing (summit disease)	Hoover et al. (2011)
*Spodoptera exigua* nucleopolyhedrovirus (SeMNPV)	Beet armyworm (*Spodoptera exigua*, Hübner)	The *egt* gene	Alters molting hormone (20-hydroxyecdysone) and infection prolong the host life span and extend its feeding period	Han et al. (2015a)
*Autographa californica* multiple nucleopolyhedrovirus (AcMNPV)	Cabbage looper (*Trichoplusia ni*, Hübner) *S. exigua*	The *egt* gene is not required for summiting	Climbing behavior in these larvae seems to be linked to molting rather than other biological processes	Ros et al. (2015)
HearNPV	Cotton bollworm (*Helicoverpa armigera*, Hübner)	The combination of the *egt* gene and unknown mechanism	20E levels decreased, but juvenile hormone (JH) titers have been shown to increase	Zhang et al. (2018)
*Bombyx mori* nucleopolyhedrovirus (BmNPV)	Silk moth (*Bombyx mori*, Linnaeus)	Protein tyrosine phosphatase (*ptp*)	*ptp* plays a crucial role in virus infection of brain tissues	Katsuma et al. (2012)
AcMNPV	*S. exigua*	*ptp* virus particles were detected in the CNS	*ptp* is needed for the expression of hyperactivity	Gasque et al. (2024)
SeMNPV	*S. exigua*	Unknown mechanisms	An attraction to light (positive phototaxis) and likely acting to promote summiting/elevation seeking	Van Houte et al. (2014)
HearNPV	*H. armigera*	Unknown mechanisms	-	Bhattarai et al. (2018a)
LdMNPV	*L. dispar*	Phototransduction and circadian entrainment pathways	An attraction to light (positive phototaxis) and likely acting to promote summiting/elevation seeking	Bhattarai et al. (2018b)
AcMNPV	*T. ni*	Unknown mechanisms	Light-independent behavior (negative geotaxis)	Han et al. (2015b)
HearNPV	*H. armigera*	Light receptor genes (*HaBL* and *HaLW*) and transient receptor potential-like channel (*TRPL*) gene	Light-induced summiting behavior	Liu et al. (2022)
HearNPV	*H. armigera*	Metabolism, immunity, and insulin signaling pathways	Infected larvae treated with insulin resulted in reduced viral replication and climbing behavior	Tian et al. (2023)
SeMNPV	*S. exigua*	Neuropeptide proctolin; *ptp* gene involvement in CNS infection	CNS, muscle contractions, and motor functions	Llopis-Giménez et al. (2022)
**Fungus**				
*Ophiocordyceps* spp.	Carpenter ants (Camponotini tribe)	Enterotoxins, Small Secreted Proteins (SSPs), Alkaloid compounds	Indirect neuromodulation: CNS and muscle tissue	de Bekker et al. (2015); Elya (2024), Fredericksen et al. (2017), Hughes et al. (2011), Mangold et al. (2019),Will et al. (2020)
*Entomophthora muscae*	Various dipterans (true flies)	Direct growth in nervous tissue, Mechanical alterations, Sesquiterpenes, visual cues, endogenous pheromones, Direct neuromodulation; Brain tissue and attraction of healthy hosts	Direct neuromodulation: Brain tissue	Bekker et al. (2021), Elya et al. (2023), Krasnoff et al. (1995), Naundrup et al. (2022)
*Pandora* spp.	Ants (*Formica* spp., Linnaeus)	Rapid tissue colonization, Mechanical manipulation	Mechanical alteration: Muscle tissue	Csata et al. (2021)
*Eryniopsis lampyridarum*	Goldenrod soldier beetles (*Chauliognathus pensylvanicus*, DeGeer)	Mechanical factors likely responsible for wing-raising behavior	Mechanical alteration: Musculature	Steinkraus et al. (2017)

### Behavior-modifying entomopathogenic *fungi*

**Fig. 3:**
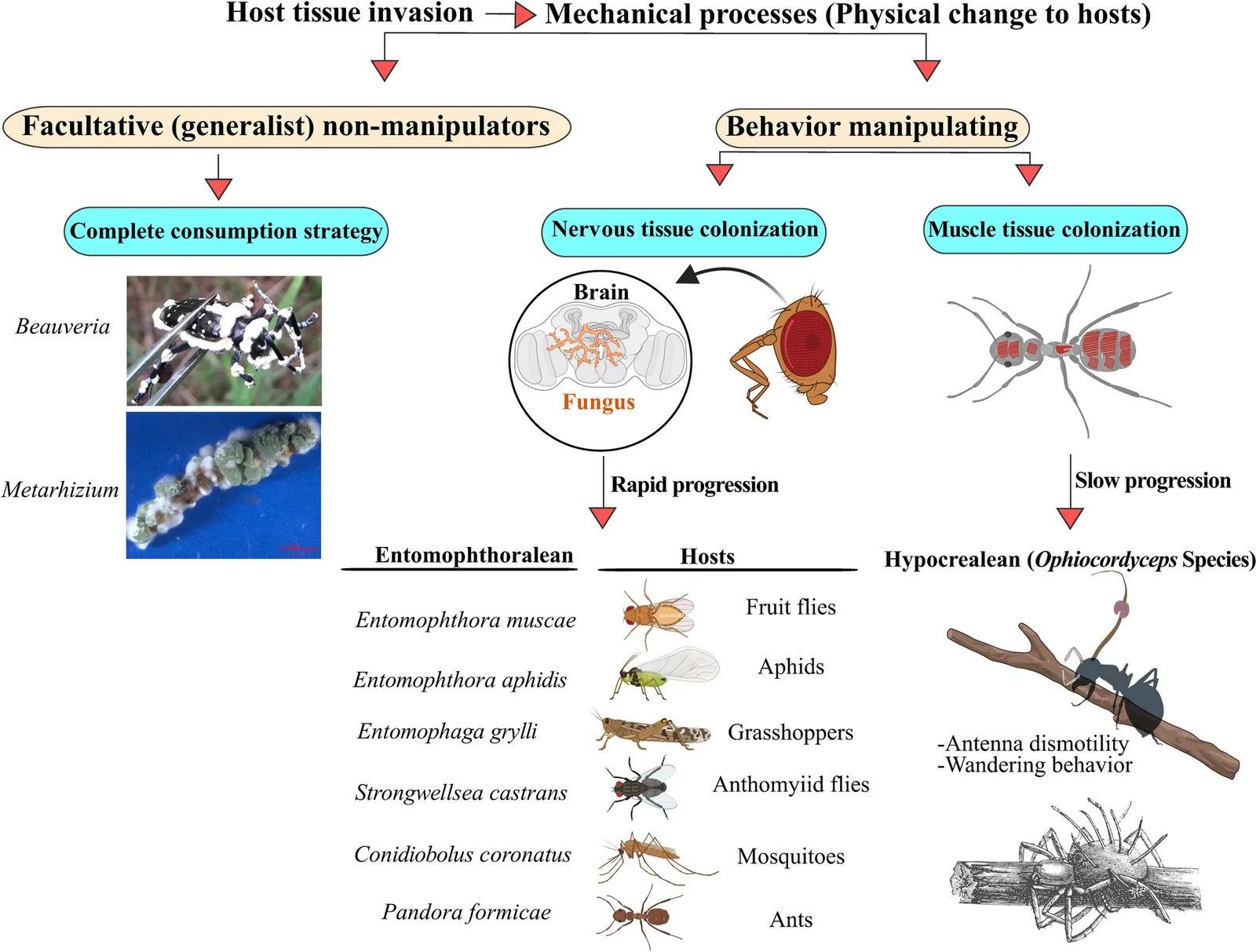
Mechanical processes in host manipulation by facultative (generalist) non-manipulators and specialized behavioral manipulating (“zombie-making”) fungi. Generalists such *Metarhizium* (*Hypocreales*, *Clavicipitaceae*) and *Beauveria* sp. (*Hypocreales*, *Cordycipitaceae*) follow a complete consumption strategy. Entomophthoralean fungi often cause rapid progression of symptoms and manipulation, whereas Hypocrealean fungi (*Ophiocordyceps* species infect different hosts, such as ants and spiders) exhibit a slower progression. Images: infected Asian long-horned beetle (*Anoplophora glabripennis*, Motschulsky) with *B. bassiana* and infected yellow mealworm (*Tenebrio molitor*, Linnaeus) larvae with *Metarhizium* (Masoudi et al. 2020)

Entomopathogenic fungi employ diverse strategies to exploit insects for nutritional purposes. These fungi typically infect insects through direct contact (*per cutaneous*), but some can also infect hosts via oral routes (*per os*) (Mannino et al. 2019). Cuticular or topical infection begins when fungal spores attach to the insect cuticle (Holder and Keyhani 2005), which then germinate and, depending upon the species, produce penetrating hyphae or appressoria, which breach the cuticle via enzymatic degradation and mechanical pressure (Ortiz-Urquiza and Keyhani 2013; Pedrini et al. 2013). Once the integument is breached, the fungus elaborates yeast-like cells termed hyphal bodies that are free-floating within the hemocoel, proliferating on hemolymph and surrounding tissue nutrients (Lewis et al. 2009; Wanchoo et al. 2009). As the insect succumbs to the infection, the fungus reverts to hyphal growth to work its way out of the insect body to grow and sporulate on the insect cadaver in processes that include suppression of competing microbes both during sporulation and by conidia as they initially infect hosts (Fan et al. 2017; Shang et al. 2024; Tong et al. 2020). Some entomopathogenic fungi are generalists, capable of infecting a wide range of host species (e.g., members of the *Beauveria*, *Metarhizium*, and *Isaria* genera) (Masoudi et al. 2018; Ortiz-Urquiza and Keyhani 2016). However, infection by these generalists typically does not result in significant manipulation of host behavior as defined herein. Typically, it is specialist entomopathogenic fungi, whose members typically target a limited array of host species, that engage in host behavioral manipulation. These fungi have been popularly referred to as “zombie-inducing fungi,” which are predominantly found in the fungal phyla *Ascomycota* (most in the order *Hypocreales*, with the members of the *Ophiocordyceps* genera being the most widely studied) and within the *Zoopagomycota* (most in the order *Entomophthorales*) (Bekker et al. 2021; Elya 2024), and aspects of their natural history have been documented in the scientific literature since the mid-nineteenth century (Thaxter, 1888). Such zombie-inducing fungi generally employ one of two strategies to access new hosts: (i) cadaver transmission or (ii) active host transmission (Lovett et al. 2020a). In cadaver-transmitting systems, the fungus manipulates the host such that death occurs in an elevated location, at which time the fungus grows outwards from the host and sporulates, utilizing the high vantage point to disperse spores. In this case, transmission occurs to the host from dead infected members. Examples of these include *Ophiocordyceps* spp., the zombie ant fungus that infects carpenter ants (Petch 1937) and *Entomophthora muscae* infects various dipterans (true flies) (Krasnoff et al. 1995). Active host transmission involves horizontal transmission of the fungus from one (living) infected member to non-infected hosts (Lovett et al. 2020a). Examples include *Massospora* infection of periodical cicadas, in which the fungus grows extensively on the lower abdomen of the host and is transmitted to other hosts via contact/sexual activity (Cooley et al. 2018). Such fungal-insect behavioral manipulation and colonization strategies include mechanical and chemical mechanisms (Fig. 3). Mechanical manipulation encompasses physical alterations such as tissue-specific colonization, changes in internal (insect) physiology, and targeted spore production, with chemical manipulation involving the production of fungal biomolecules that disrupt and control host cellular, immunological, and neurological processes (Bekker et al. 2021; de Bekker and Das 2022). Generally, two basic chemical signaling mechanisms implicated in behavior manipulation by zombie-making fungi have been distinguished: (i) direct neuromodulation, where fungal factors act directly on neural circuits, and (ii) indirect neuromodulation, where fungal factors alter upstream inputs to neural circuits. Hypocrealean fungi (e.g., *Ophiocordyceps*) do not appear to directly invade host nervous tissue, suggesting an indirect mechanism of CNS manipulation. In contrast, many Entomophthoralean fungi (e.g., *Entomophthora*) directly grow in host nervous tissues (Bekker et al. 2021) and colonize brain tissues while the host is still alive (Bekker et al. 2021; Brobyn and Wilding 1983; Csata et al. 2021; Elya 2024; Elya et al. 2018; Funk et al. 1993). Conversely, *Ophiocordyceps* species invade the nervous tissue after host death despite their extensive colonization of muscle tissue (Fredericksen et al. 2017; Hughes et al. 2011; Mangold et al. 2019). These two groups also exhibit different rates of disease progression. *Ophiocordyceps* infections progress slowly over several weeks, allowing a greater length of time for behavioral manipulations (2017a; de Bekker et al. 2015; Will et al. 2020). In contrast, *Pandora* (*Entomophthora*, *Entomophthoraceae*) species colonize internal tissues of their ant hosts within days, leading to behaviors including mound biting and/or gripping of grass blades near the ant mound, typically occurring several days post-infection. The exact timing of these behaviors can vary depending on environmental factors like temperature and humidity, as well as the specific fungal *Pandora* species and the ant host involved (Csata et al. 2021).

Behavioral manipulation by entomopathogenic fungi can include various combinations of hyperactivity, gravitropism (summiting), phototaxis, dying-phase positional adjustments (DPPA), circadian timing, and/or sexual attraction (Elya 2024). Hyperactivity typically precedes summitting and is a hallmark of several fungal insect pathogens, particularly within the *Ophiocordyceps* genera (Hughes et al. 2011) as well as those observed in *Entomophthora muscae* infections (Krasnoff et al. 1995), and is one of the most consistently observed behavioral patterns in these cadaver-transmitting fungal parasites. Field studies have shown that *Ophiocordyceps*-infected ants are more likely to die in sunlit areas than in shaded ones (Andriolli et al. 2019), and their heads (once dead) are often oriented toward canopy openings (Chung et al. 2017). As summit behavior involves climbing, these fungi disrupt normal neuronal responses via mechanisms that differ from their viral counterparts.

Fungal-induced hyperactivity likely arises from general physiological changes that may include starvation-like states induced as the fungi proliferate within the host, leading to increased locomotion (Elya et al. 2018; Will et al. 2020). Hyperactivity may be driven by compounds secreted by the fungus, including enterotoxins and small secreted proteins (SSPs) that have been shown to be produced, for example, by *Ophiocordyceps* during infection of host ants. Enterotoxins may disrupt host chemosensory functions (de Bekker et al. 2017a), and SSPs are believed to influence host behavior by affecting neurobiological processes (Bekker et al. 2021). This is supported by gene expression data which has shown that expression of enterotoxin and SSPs genes, as well as a gene cluster responsible for the synthesis of the alkaloid compound related to the tremorgenic compound, aflatrem, remains high during the biting phase induced by *Ophiocordyceps*, which represents the penultimate step of the infection (before sporulation) as summiting peaks (de Bekker et al. 2015; Elya 2024; Will et al. 2020). However, genetic confirmation and/or direct testing of the roles of these proteins remain to be performed. Insects infected by behavior-manipulating fungi often display significant DPPA (dying-phase positional adjustments), with positioning/fixing of mouthparts (on leaves, twigs, grass blades, and other substrates) and the splaying of legs and wings (Elya 2024), which occur after ascension to elevated positions, and represents a behavior hallmark of cadaver-transmitting infection mechanisms (Bekker et al. 2021). For instance, *Ophiocordyceps*-infected ants exhibit “lockjaw,” where they bite onto a substrate before death, facilitated by fungal structures penetrating their muscles (Fredericksen et al. 2017; Hughes et al. 2016; Mangold et al. 2019). Similarly, *Entomophthora*-infected flies extend their proboscis onto their perched substrate, becoming “glued” to place by fungal secretions (Brobyn and Wilding 1983). This proboscis extension might be mechanically driven by body cavity pressure and/or influenced by fungal interactions with motor neurons (Elya et al. 2018). Such positioning and fixation are influenced by circadian clock/timing and/or, in some instances, signals/attraction aimed towards healthy, uninfected hosts. For example, after summiting, both *E. muscae*-infected flies and *Eryniopsis lampyridarum* (*Entomophthorales*, *Entomophthoraceae*) infected goldenrod soldier beetles (*Chauliognathus pensylvanicus*, DeGeer) elevate their wings presumably to facilitate spore dispersal from the dorsal abdomen. The rapid wing-raising in flies (completed in about 15 min) may result from fungal growth impinging on musculature and/or alterations in motor neuron activity (Krasnoff et al. 1995). Infected soldier beetles exhibit a slower wing-raising process, with mechanical factors likely responsible (Steinkraus et al. 2017). Interestingly, similar DPPAs are observed in insects parasitized by non-fungal organisms. Ants infected by the trematode *D. dendriticum* also maintain elevated positions by biting down, though they remain alive for several days to increase the likelihood of being consumed by herbivores, benefiting the parasite by transferring it to its next host (Martín-Vega et al. 2018). The specific mechanisms behind this behavior in trematode-infected ants remain unclear, although the presence of trematode flukes in the subesophageal ganglion (SOG) might play a role (Martín-Vega et al. 2018).

“Zombie” fungi commonly induce behavioral changes and subsequent death in their hosts as a function of light/dark circadian rhythms. Flies infected with *E. muscae* exhibit summit disease and DPPAs, specifically at sunset (Elya et al. 2018; Krasnoff et al. 1995), while *Ophiocordyceps*-infected ants summit and die within a 3-h window at dawn or in the mid-late morning (solar noon), depending on the species (Hughes et al. 2011; Westwood et al. 2019). This timing involves the interaction of two molecular clocks: one from the host and one from the fungus. Molecular clocks can maintain circadian rhythms, in part, independent of environmental cues, and are driven by a set of core transcription/translation feedback loops that have been observed in some form in almost all eukaryotic organisms (Doležel 2023; Dunlap and Loros 2017). In *E. muscae*-infected flies, the timing of death appears to be driven by the fungal rather than the host clock. Evidence for this includes the lack of rhythmic timing of death in flies infected under constant darkness, despite the host maintaining circadian periodicity in such conditions (Krasnoff et al. 1995). Additionally, flies exposed to darkness 72-h post-infection show rhythmic timing of death, indicating that the fungal machinery can synchronize its internal clock with the host’s environment after the infection has begun. This synchronization process, known as entrainment, allows the fungus to adjust its circadian rhythm to match external cues, ensuring that the timing of critical events, such as host death, maximizes completion of the fungal life cycle and spore dispersal (Krasnoff et al. 1995). Although the presence of a fungal clock in *E. muscae* is likely, it has not been conclusively demonstrated. In *Ophiocordyceps*-infected ants, timing is hypothesized to be manipulated by fungal influence/interference of the host clock. Transcriptomic studies revealed perturbations in the expression of various clock genes in *Ophiocordyceps* parasitism of ants during the infection process (de Bekker et al. 2015; Will et al. 2020), with infected ants losing their typical foraging rhythms (Trinh et al. 2021). Expression of circadian transcription patterns in *Ophiocordyceps* when grown in vitro on media in the absence of the host has confirmed that the fungus has its own molecular clock (de Bekker et al. 2017b). These data suggest that *Ophiocordyceps* exhibits gene expression cycles that follow an approximate 24-h period, even without external environmental cues such as light and temperature changes. The exact mechanisms linking fungal clock activity to host behavior remain unclear, but it is proposed that the fungus secretes compounds that alter host physiology, potentially affecting neuronal populations or triggering internal state changes that lead to altered behaviors (de Bekker and Das 2022).

The critical aspect of summit disease is for the pathogen to find the next host. Expelling spores to “rain” down on unsuspecting hosts is one means; however, conceptually, the ability to (additionally) lure healthy hosts to the fungus would provide significant advantages and would be highly adaptive. In support of this, it has been shown that *E. muscae* enhances transmission by attracting healthy hosts to infected flies. Male house flies are drawn to late-stage female cadavers, with a number of potential factors contributing to this attraction, including via (i) fungal-produced compounds (notably sesquiterpenes), (ii) visual cues from the death pose that may mimic sexual availability, and/or (iii) fungal-induced production of endogenous female pheromones (Naundrup et al. 2022). Since the host is dead, this attraction clearly benefits the fungus and mirrors the activities of other fungi that employ chemical mimicry to attract hosts, e.g., the luring of *Caenorhabditis elegans* nematodes (Rhabditida) by the predatory fungus *Arthrobotrys oligospora* (Orbiliales, Orbiliaceae) (Hsueh et al. 2017) and by the pathogenic bacterium *B. nematocidal* (Bacillales, Bacillaceae) (Zhang et al. 2020).

Overall, while these behavioral changes are seen to benefit the parasites, it is possible that some of these behaviors exhibited by hosts (if still alive) are attempts to increase (or at least engage in) reproductive output before death, avoid spreading the infection to healthy conspecifics, and/or fight disease progression. With respect to reproductive output, in many cases, because of infection, the host is rendered sterile; thus, it is unclear whether such behaviors would represent engagement in futile reproductive efforts or another aspect of the pathogen-host manipulation. In addition, it is known that some fungal insect pathogens, e.g., *Massospora* sp., actively promote (increased) sexual activity/contact as a mechanism for their horizontal transfer to healthy hosts; however, these fungi do not cause summit disease (Lovett et al. 2020a). A summary of the major fungal insect pathogens resulting in summit disease is included in Table 1.

## Summit disease and insect neuroanatomy

**Fig. 4:**
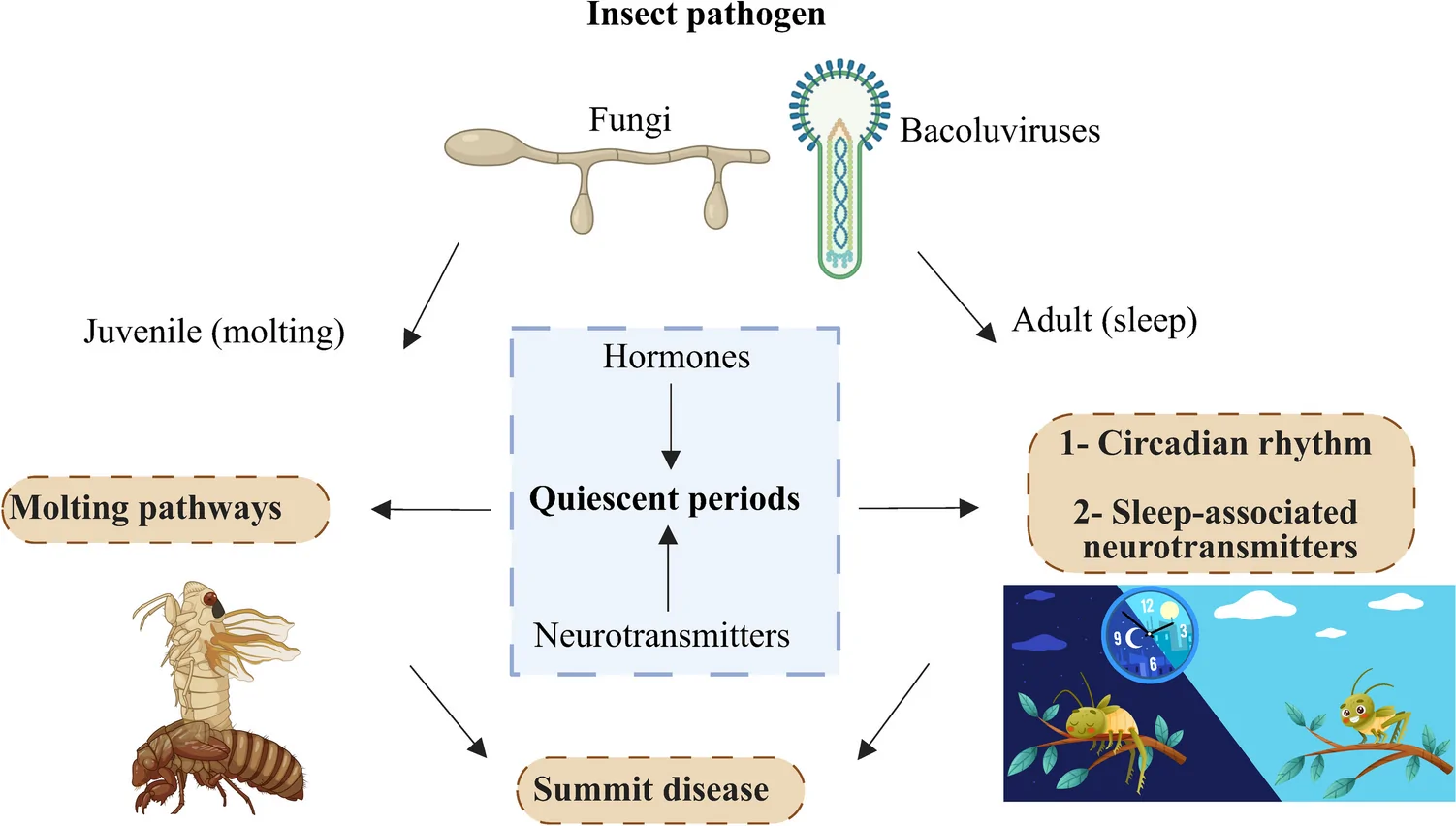
Types of summit disease: juvenile and adult, exploiting behaviors of infected insects during periods of quiescence. Image adapted from (Lovett et al. 2020b)

**Fig. 5:**
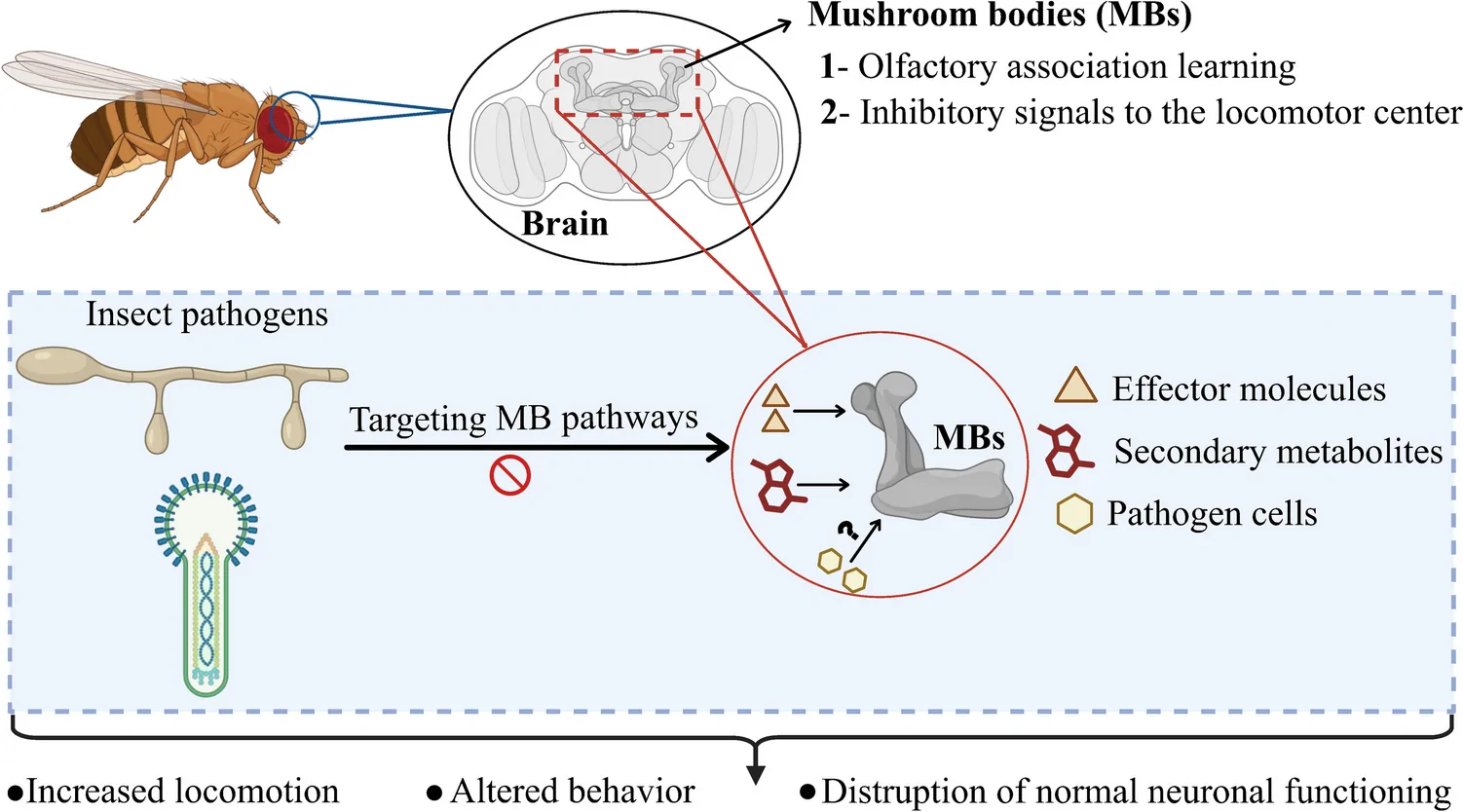
Simplified potential general mechanism of enhanced insect locomotion through pathogen targeting of mushroom bodies (MBs). Targeting of MBs can alter olfactory association learning and inhibitory signals to the locomotor center, resulting in increased locomotion. The neuroanatomy of the sequence of *E. muscae*-induced summiting of host flies on a much more refined scale is presented in Elya et al. (2023)

Summit disease represents an example of the convergent evolution of an extended phenotype by phylogenetically diverse pathogens. As such, these viral and fungal insect pathogens have evolved independently to manipulate sequential behavioral levers in their respective hosts. However, as part of this selective evolutionary process, these pathogens may have exploited ancestral behaviors shared by their respective hosts (Lovett et al. 2020b). These shared behaviors have been suggested to be (i) periods of quiescence during molting in immature insects and/or (ii) sleeping in adult insects, both of which are highly conserved across many insect species. In this context, a distinction between two types of summit disease, juvenile and adult, has been proposed (Fig. 4). In insects, the putative sleep–wake centers are located in higher-order brain centers and are indirectly connected to the circadian clock network (Helfrich-Förster 2018). Structures termed mushroom bodies (MBs) found in the insect brain are considered to be crucial areas responsible for cognitive processing, including integrating mental and sensory inputs (Rubin and Aso 2024). MBs are essential for olfactory association information, walking-related locomotion, and predatory/enemy avoidance (Smith and Lei 2023), and enable insects to adapt to their environments, remember past experiences, and execute complex behaviors (Lin 2023). Intriguingly, blockage of MB activity leads to an increase in locomotor activity in crickets and grasshoppers (Orthoptera) (Huber 1960; 1974). Similarly, male fruit flies exhibit increased activity levels after their MBs were destroyed using hydroxy urea (Helfrich-FÖRster et al. 2002). In addition, fruit flies without functional MBs are not only more active but also sleep less than control flies (Joiner et al. 2006). MB neurons affected by serotonergic and GABAergic neurons (gamma-aminobutyric acidergic) promote wakefulness and are inhibited by either GABA, serotonin, or both. Consequently, activating these serotonergic neurons has a strong sleep-promoting effect (Haynes et al. 2015). This finding is consistent with evidence showing that fruit flies with a loss of 5-hydroxytryptamine (d5-HT1) serotonin receptors in their MBs experience less sleep (Yuan et al. 2006). In a series of elegant experiments using a high-throughput approach to measure summiting, the increased locomotory activity in *E. muscae*-infected flies was shown to integrate host circadian responses with specific neurosecretory systems, that included the DN1p circadian neurons, the pars intercerebralis to corpora allata projecting (PI-CA) neurons, and the corpora allata (CA, site of JH synthesis) (Elya et al. 2023). Further analyses showed that PI-CA neurons and the CA were intact in summiting animals, despite other regions of the brain (and the insect as a whole) showing fungal penetration and proliferation. Increased permeabilization of the blood–brain barrier of flies was noted during the infection progression and transfusion of fungal-infected fly hemolymph into non-summiting flies resulted in a “burst of locomotion.” These data coupled to potential life-stage parameters suggest that irrespective of the insect life stage, insect-behavior modifying pathogens (in summiting and beyond) target a range of host brain structures, potentially centered on MB neurons, which may account for generalized phenotypes of hyperactivity and less sleep (Fig. 5). Understanding if and how these fungal pathogens and their distinct but related “extended phenotypes” target MB neurons to modify insect behavior will provide insights into the shared and unique neurological mechanisms behind how these varied pathogens “control” their different hosts. Furthermore, identifying the commonalities and differences in how circadian rhythms are manipulated in summit diseases across various stages and pathogen systems will allow for a more comprehensive understanding of behavior-manipulating phenotypes (Westwood et al. 2019).

## Future directions

Advancing the understanding of summit disease and pathogen-induced behavioral manipulation in insects requires investigating both the natural history and molecular mechanisms of the process. As the systems that result in summit disease are widely disparate (viral to fungal), it is possible that a unified theory is not possible and that these are essentially different diseases that share a superficial commonality. Conversely, considering constraints on host physiology (neuronal functioning), even though the (parasite molecular) mechanisms may be unrelated, the (host) targets may be shared. This insight could allow for the integration of various summit disease systems with their manipulated behavioral outputs. We propose that such a nexus, affecting hyperactivity, phototaxis, and gravitaxis, via targeting/neuromodulation of MBs (and other brain structures) may be a common feature of summit disease caused by insect pathogens. Research examining the functioning and targeting of MBs during infection is warranted. In addition, coupling of cell physiology/biology with molecular manipulation of pathogen genes implicated in altering neural circuitry and neurotransmitter dynamics is needed. Such functional genetics, including global gene expression responses (in both the host and the pathogen) coupled to metabolomics, should also be employed to examine host processes induced during summit disease progression with attention to circadian rhythms and neurological processes. Additional aspects that remain largely unexplored include host immunological effects, including in brain (immune) cells, effects/interactions with insect endosymbionts and cuticular and gut microbiome, resistance mechanisms on the host, and the nature of the host specificity of most summit disease-causing insect pathogens.
